# Quantitative Imaging Informatics for Cancer Research

**DOI:** 10.1200/CCI.19.00165

**Published:** 2020-05-11

**Authors:** Andrey Fedorov, Reinhard Beichel, Jayashree Kalpathy-Cramer, David Clunie, Michael Onken, Jörg Riesmeier, Christian Herz, Christian Bauer, Andrew Beers, Jean-Christophe Fillion-Robin, Andras Lasso, Csaba Pinter, Steve Pieper, Marco Nolden, Klaus Maier-Hein, Markus D. Herrmann, Joel Saltz, Fred Prior, Fiona Fennessy, John Buatti, Ron Kikinis

**Affiliations:** ^1^ Department of Radiology, Brigham and Women’s Hospital, Harvard Medical School, Boston, MA; ^2^ University of Iowa, Iowa City, IA; ^3^ Massachusetts General Hospital, Boston, MA; ^4^ PixelMed Publishing, Bangor, PA; ^5^ OpenConnections, Oldenburg, Germany; ^6^ Freelancer, Oldenburg, Germany; ^7^ Kitware, Clifton Park, NY; ^8^ Queen’s University, Kingston, ON, Canada; ^9^ Isomics, Cambridge, MA; ^10^ German Cancer Research Center, Heidelberg, Germany; ^11^ Department of Pathology, Massachusetts General Hospital, Harvard Medical School, Boston, MA; ^12^ Stony Brook University, Stony Brook, NY; ^13^ University of Arkansas for Medical Sciences, Little Rock, AR

## Abstract

**PURPOSE:**

We summarize Quantitative Imaging Informatics for Cancer Research (QIICR; U24 CA180918), one of the first projects funded by the National Cancer Institute (NCI) Informatics Technology for Cancer Research program.

**METHODS:**

QIICR was motivated by the 3 use cases from the NCI Quantitative Imaging Network. 3D Slicer was selected as the platform for implementation of open-source quantitative imaging (QI) tools. Digital Imaging and Communications in Medicine (DICOM) was chosen for standardization of QI analysis outputs. Support of improved integration with community repositories focused on The Cancer Imaging Archive (TCIA). Priorities included improved capabilities of the standard, toolkits and tools, reference datasets, collaborations, and training and outreach.

**RESULTS:**

Fourteen new tools to support head and neck cancer, glioblastoma, and prostate cancer QI research were introduced and downloaded over 100,000 times. DICOM was amended, with over 40 correction proposals addressing QI needs. Reference implementations of the standard in a popular toolkit and standalone tools were introduced. Eight datasets exemplifying the application of the standard and tools were contributed. An open demonstration/connectathon was organized, attracting the participation of academic groups and commercial vendors. Integration of tools with TCIA was improved by implementing programmatic communication interface and by refining best practices for QI analysis results curation.

**CONCLUSION:**

Tools, capabilities of the DICOM standard, and datasets we introduced found adoption and utility within the cancer imaging community. A collaborative approach is critical to addressing challenges in imaging informatics at the national and international levels. Numerous challenges remain in establishing and maintaining the infrastructure of analysis tools and standardized datasets for the imaging community. Ideas and technology developed by the QIICR project are contributing to the NCI Imaging Data Commons currently being developed.

## INTRODUCTION

Medical imaging is increasingly important in cancer applications.^1,2^ Few existing imaging biomarkers are used to guide clinical decisions.^3^ Major efforts are underway to identify, validate, and deploy new imaging tools in the clinic. These efforts rely on the discovery of novel quantitative imaging (QI) biomarkers, which promise to support objective and reproducible characterization of disease and allow for more personalized approaches to diagnosis and therapy. One such effort is led by the National Cancer Institute (NCI) via its Quantitative Imaging Network (QIN) initiative,^4-6^ with primary goals including collecting data from ongoing imaging clinical trials, developing innovative methods for data collection and analysis, and establishing consensus on QI methods.^4^

**CONTEXT**

**Key Objective**
To present the motivation, approach, results, and vision for the future development for the Quantitative Imaging Informatics for Cancer Research project, funded by the National Cancer Institute Informatics Technology for Cancer Research program.
**Knowledge Generated**
Free, commercially reusable, open-source tools were developed by the project to support imaging informatics tasks related to quantitative image analysis standardized communication of analysis findings. Contributions to the Digital Imaging and Communications in Medicine (DICOM) standard, collaborations, community engagement activities, and public datasets contributed by the project are summarized.
**Relevance**
Our contributions can be used to support additional technical and clinical validation of imaging biomarkers, and improve harmonization of clinical and research imaging data. We also set the stage and methodology for future projects to further improve capabilities, support, and adoption of the DICOM standard in imaging research, radiomics, and machine learning.

Practical QIN experience in striving toward those goals highlighted the importance of imaging informatics as applied to QI biomarker development. In the context of radiology, imaging informatics is defined as a subspecialty of radiology concerned with “the study of how information about medical images is exchanged within radiology and throughout the medical enterprise”^7^ ^(p657)^. Communication of health care information and integration of data from various institutions and sources is critical for development and validation of innovative QI research tools. Research tools need to be harmonized to support data interoperability, reuse, and aggregation in community repositories and enable federated learning and eventual translation of the matured tools into clinical trials. The Informatics Technology for Cancer Research (ITCR) program (https://itcr.cancer.gov/) was established by NCI to support development and sustainment of open-source cancer informatics tools spanning all areas of cancer research.

Quantitative Imaging Informatics for Cancer Research (QIICR; U24 CA180918) was one of the first projects funded by ITCR under PAR-12-287. Motivated by challenges encountered by the QIN community, and with several investigators actively or formerly funded by the QIN program, QIICR embarked on a mission to close some of the informatics gaps that were perceived as limitations to the success of QIN.

The QIICR project developed a collection of reusable open-source components to support a range of informatics tasks for QI. In parallel, we worked on improving standardization of QI analysis outputs by refining and improving the existing standard, developing reference tools and datasets implementing the standard, and performing various outreach activities to demonstrate and promote best practices for sharing QI analysis results. We summarize the scope, collaborative approach, vision, and results of the QIICR project over the funded period; discuss the legacy of the project in the context of various ongoing activities; and outline remaining challenges and opportunities that we identified.

## METHODS

The QIICR approach was defined by the three aims:

1.establishing open-source tools and workflows to support analysis of longitudinal and derived image data;
2.supporting standardized representations for communicating QI analysis results; and
3.improving interoperability with community image data repositories.

Three collaborating QIN groups joined the QIICR consortium and defined clinical applications, imaging modalities, analysis approaches, and generated data types. They also contributed to the development of tools and supplied representative datasets:

1.Quantitative magnetic resonance imaging (MRI) of glioblastoma treatment response (U01CA154601; J.K.-C. at Massachusetts General Hospital);
2.Quantitative positron emission tomography (PET)/computed tomography (CT) of head and neck cancer (U01CA140206; J.B. and R.B. at the University of Iowa);
3.Multiparametric MRI of prostate cancer (U01CA151261; F.F. at Brigham and Women’s Hospital [BWH]).

We committed to share all software tools under a commercially friendly, nonrestrictive software license to facilitate reuse, adoption, and external contributions, without limiting them to the QIN community. 3D Slicer, an extensible open-source platform for imaging research,^8^ was selected as a delivery vehicle for end-user capabilities. The goal of the project was to produce open-source implementations of the application-specific analysis tools and processing workflows within 3D Slicer, while also making it feasible to use those tools outside of 3D Slicer and in applications beyond the scope of the QIICR project.

To harmonize the various outputs generated by QI research analysis tools, we used the Digital Imaging and Communications in Medicine (DICOM) standard.^9^ DICOM defines standard information objects to represent a broad range of acquired and image-derived data and is suitable for clinical, preclinical, and research applications. DICOM is a comprehensive and complex standard, used within the research community primarily as a mechanism for receiving images from clinical systems. Capabilities of DICOM beyond its support of clinical imaging modalities are not well known to researchers. In a typical research workflow, DICOM images obtained from clinical systems are converted into research formats supported by commonly used analysis tools. This approach allows for limited interoperability between specific research tools, but leads to difficulty reusing analysis results for other purposes. Most research formats do not communicate standardized metadata describing the dataset, do not contain metadata that identifies and relates individual objects, and do not follow a common data model. Standard concepts for identification of the subject, dates, unique identifiers, and spatial or temporal frames of reference are missing. Translation of QI tools into clinical trials, and evaluation of their use for decision support in clinical practice, is a key priority for the QIN. Integration of research tools and the data they generate necessitates the use of an established standard such as DICOM. Our approach identified the types of analysis results that were relevant to the QIICR use cases and remediated the gaps related to use of DICOM for those results along the following directions:

1.Refinements and extensions: We evaluated existing capabilities of the standard to understand how those related to the requirements of the QIN use cases, which needs could be addressed by improving documentation or implementing support in tools and toolkits, and which required enhancements to the standard.
2.Implementation of the relevant capabilities: To encourage and simplify adoption, we improved support of the relevant components of DICOM in existing open-source tools, developing new tools as necessary.
3.Publicly available reference datasets: To evaluate the capabilities of both the standard and its implementations, to support future adopters, and to demonstrate our recommended approach to deriving data curation via concrete examples, we worked on encoding relevant datasets.
4.Training and outreach: To lower barriers to adoption, we contributed scholarly publications, training materials, and tutorials explaining the best practices for using DICOM and the tools we developed. We prioritized collaborations with other groups (academic and commercial) developing imaging analysis tools and workstations to encourage, support, and evaluate adoption of DICOM.

For the third aim, we directed our effort to improved integration with The Cancer Imaging Archive (TCIA),^10^ the recommended repository for the QIN investigators. TCIA implements procedures that ensure DICOM imaging data are free from protected health information and maintains resources to support sharing of de-identified data.^11^ Increasingly, TCIA is also distributing analysis results alongside the image data. We aimed to simplify the process of interaction of tool users with the repository and worked on DICOM conversion of the QI analysis results in other formats already deposited in TCIA.

## RESULTS

### Quantitative Image Analysis Tools

**FIG 1.**
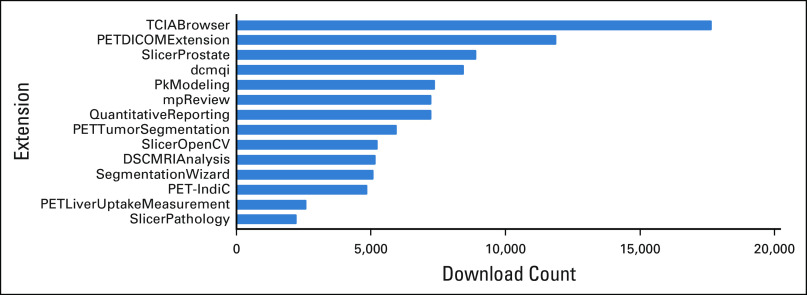
Download statistics for the 3D Slicer extensions developed with the contribution of the Quantitative Imaging Informatics for Cancer Research (QIICR) project. Note that extensions were released at different times, with some extensions being available longer than others. Some of the extensions are dependent on others (eg, PETDICOMExtension can be installed individually, but is also downloaded every time PET-IndiC or QuantitativeReporting is installed). Statistics reported were collected on December 15, 2019, using the script available publicly at https://github.com/Slicer/SlicerDeveloperToolsForExtensions. These reported download counts do not include downloads of the binary packages directly from GitHub or downloads of Docker images from DockerHub (which were used for disseminating dcmqi packages), or downloads of the source code (due to technical limitations of being able to track such downloads).

A range of batch-processing and interactive tools were developed to support QI workflows for each of the use cases. Batch-processing C++ Slicer Execution Model (SEM) plugins were added for 3D Slicer.^8^ These modules can be used by other applications adopting SEM.^12,13^ The plugins can also be used as standalone command-line tools outside of 3D Slicer. Interactive tools were implemented as 3D Slicer Python scripted modules. Modules were disseminated using the 3D Slicer extensions infrastructure, which supports automatic testing and packaging of extensions for Windows, macOS, and Linux. The tools are summarized in Table 1 and Figure 1 and on the QIICR Web site.^14^

**TABLE 1.**
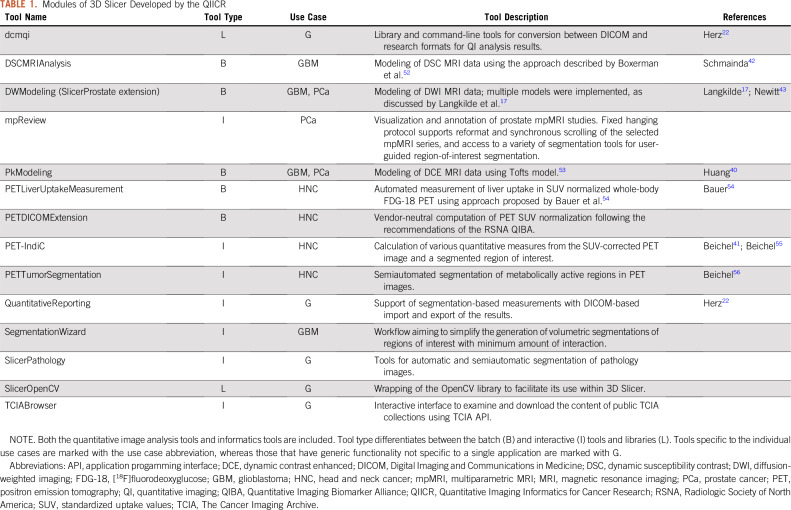
Modules of 3D Slicer Developed by the QIICR

We engaged in a number of efforts led by QIN to evaluate the developed tools using common reference datasets and to compare the results produced by the various tools available within the network (Table 1). We also conducted a number of studies designed to develop best practices in corresponding QI methodologies.^15-19^

### Standardized Representation of QI Analysis Results

To enhance the DICOM standard based on the needs of the use cases, we contributed numerous improvements to the standard (some of those are discussed in detail in Fedorov et al^20^; see the Data Supplement for the complete list). Per the DICOM process, those updates passed the rigor of public review, revision, and approval by the DICOM standard committee.

Our DICOM implementation efforts improved support of relevant DICOM components in a variety of tools. We added new capabilities to the DICOM Toolkit (DCMTK) C++ library,^21^ improving read/write support and the application programming interface (API) for DICOM Segmentation and Parametric Map objects and the DICOM TID 1500 Structured Report template. The API introduced an extra level of abstraction to simplify both reading and writing of those objects. We developed *dcmqi*,^22^ a standalone library and set of command-line converters (implementing the SEM interface) between standard and research-oriented representations of the aforementioned objects. *dcmqi* has subsequently been integrated into a number of independent platforms, such as MITK^12^ and ePAD,^23^ and has supported data conversion tasks for several other projects.^24,25^ The *Quantitative Reporting* extension of 3D Slicer^22^ was developed to hide (behind the interactive user interface) the peculiarities of reading and writing QI DICOM objects.

We worked with the CommonTK, ePad, and OHIF communities to create JavaScript implementations of key DICOM processing code to use in Web browsers and other JavaScript environments. An early experiment was to cross-compile DCMTK C++ code to JavaScript, using Emscripten.^26,27^ More recently, we have been developing a pure-JavaScript suite of libraries to natively implement critical DICOM functionality, including a new version of *dcmjs* that manages conversion of traditional DICOM binary format to and from JavaScript objects and JavaScript object notation.^28^ The new *dcmjs* is also used as the core library for JavaScript implementation of client and server portions of the DICOMweb standard.^29^ These libraries have been used, for example, as part of the ITCR–Radiologic Society of North America (RSNA) Crowds Cure Cancer collaboration.^30^

**FIG 2.**
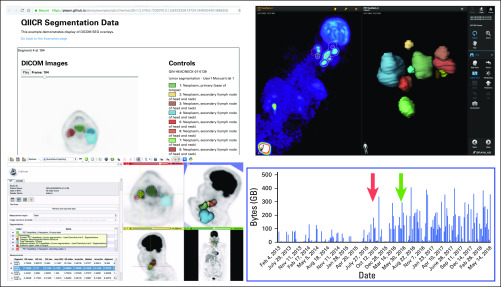
Example demonstration of image analysis results interoperability enabled by DICOM. From bottom left corner clockwise, examples of platforms visualizing the same Digital Imaging and Communications in Medicine (DICOM) positron emission tomography (PET) segmentation dataset from the public QIN-HEADNECK collection^20,61^: 3D Slicer^8^ (free, open-source desktop application), OHIF Viewer^38^ (free, open-source Web viewer), Brainlab SmartBrush (commercial Food and Drug Administration–approved tumor outlining application). Results were collected as part of the DICOM4QI (DICOM for Quantitative Imaging) demonstration and connectathon organized by Quantitative Imaging Informatics for Cancer Research (QIICR) at the annual Radiologic Society of North America meeting since 2015.^36,62^ The histogram shows the significant increase in the The Cancer Imaging Archive–reported usage of the QIN-HEADNECK collection after the publication of the preprint in Nov 2015 (red arrow) and peer-reviewed paper in May 2016 (green arrow),^20^ which introduced imaging-related DICOM data (segmentations, measurements, clinical data) to accompany the imaging dataset.

We published a number of datasets accompanied by peer-reviewed articles demonstrating best practices for QI analysis results sharing (Table 2; Fig 2). Those supported the data-sharing activities of the collaborating QIN projects and demonstrated the capabilities of the standard and the developed tools. For example, test-retest measurements^31^ obtained from an annotated prostate multiparametric MRI dataset^32^ were instrumental in defining claims in the Quantitative Imaging Biomarker Alliance Diffusion-Weighted Magnetic Resonance Imaging profile^33^ and the DCE (dynamic contrast enhanced) MRI Quantification under development; it was further used to evaluate repeatability of radiomics^34^ features in the prostate.^35^

**TABLE 2.**
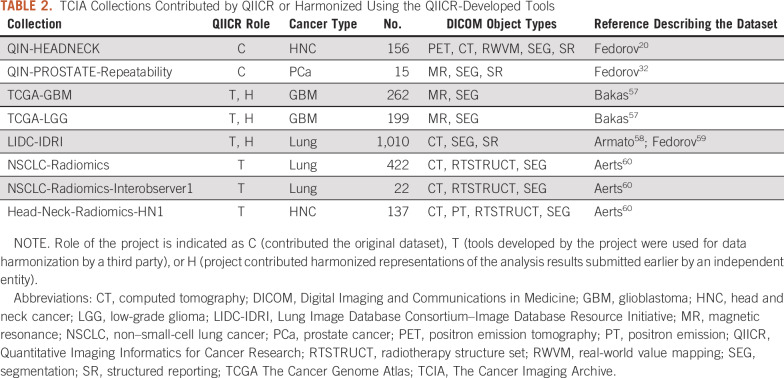
TCIA Collections Contributed by QIICR or Harmonized Using the QIICR-Developed Tools

In 2015, we initiated DICOM4QI (DICOM for Quantitative Imaging), a demonstration/connectathon project to evaluate and advance adoption of DICOM for communicating QI analysis results. DICOM4QI was an exhibit at the Quantitative Imaging Reading Room of the RSNA annual meeting. A publicly available Web site provided participation instructions and sample datasets, and summarized participation results.^36^ Over 15 platforms were included in DICOM4QI. Notably, several platforms that initially did not use suitable DICOM objects for communicating analysis results implemented that capability and used DICOM4QI as a venue for testing interoperability with other platforms. Platforms included the open-source 3D Slicer,^8,37^ MITK,^12^ ePAD,^23^ and OHIF Viewer,^38^ and commercial products such as those from Brainlab and TeraRecon (Fig 2).

We organized a half-day tutorial on the use of DICOM for QI research at the Medical Image Computing and Computer Assisted Interventions conferences in 2017 and 2018. The tutorial included presentations from QIICR investigators and collaborators, and a hands-on component demonstrating the capabilities of the DICOM standard and tools. Materials for those tutorials (presentation slides, pointers to the datasets, Jupyter notebooks) were archived and are publicly available.^39^

### Interoperability With Data Repositories

We developed *TCIA Browser,* a 3D Slicer module that uses the TCIA API to explore, download, and visualize the content of public TCIA collections. It streamlines 3D Slicer user interaction with the repository, and makes it easier to evaluate various visualization and analysis tools available within the application.

Coordinating with the TCIA team, we converted some of the existing third-party-contributed analysis results to a standard representation. These datasets can now be accessed from the usual search interface of TCIA or directly via a digital object identifier (as opposed to being linked as compressed archives from collection wiki pages) and are interoperable with a number of popular open-source tools. Furthermore, several collections were harmonized using *dcmqi* without direct involvement of the QIICR team. Table 2 lists all such collections directly or indirectly supported by QIICR. Currently, *dcmqi* is the tool recommended by TCIA for harmonizing analysis results submitted to the archive, and 3D Slicer is listed as the only visualization and analysis desktop application integrated with TCIA via its API.

## DISCUSSION

The QIICR project aimed to address major perceived needs of the QI community and the QIN, and proposed solutions for specific QIN projects. There is no clear convention on evaluating contributions such as software tools or datasets that QIICR produced. Based on the evidence we presented, at least some of the tools we developed have been used in projects external to QIICR. The MRI modeling tools and PET segmentation tools we contributed scored favorably based on the results of the challenges organized by QIN.^40-43^ Unlike most of the other tools evaluated in those challenges, QIICR-developed tools are readily available as 3D Slicer extensions; source code is public under commercially friendly, nonrestrictive, open-source license; and use of the tools does not require establishing material transfer agreements.

Our investigation of DICOM to represent QI analysis results had limited precedent in the research community. By the end of the project, we were encouraged to see increasing adoption of our ideas in both academic and commercial tools. Our contributions to the DICOM standard passed the rigor of the community review process and are now being reused in new contexts and areas of application. Through one of the QIICR collaborations, we have been investigating the use of the DICOM standard to support digital pathology applications.^44^ DICOM Working Group 23 is actively exploring the use of the DICOM TID 1500 Structured Reporting template, as well as DICOM Segmentation objects and Parametric Maps to support a host of artificial intelligence (AI) use cases, which subsume but are not limited to QI applications. We collaborated with the Imaging Biomarker Standardization Initiative (IBSI), which, among other objectives, is establishing a consensus nomenclature and definitions for radiomics features.^45^ We worked with IBSI to augment the feature definitions with codes and added corresponding coded terms to the DICOM standard (see CP-1705 and CP-1764; Data Supplement). Furthermore, we augmented *pyradiomics*,^46^ an open-source tool for extracting radiomics features, with the experimental features to accept input in DICOM format and store calculated features as DICOM Structured Reports, which use IBSI-defined codes. We consider this collaboration to be one example of successful translation of domain experts’ consensus into the standard and implemented in tools for broader deployment and adoption.

The importance of metadata and the use of standards are becoming prominent with the increasing emphasis on quality of radiomics studies^47^ and distributed learning,^48,49^ where data cannot be shared outside the institution because of privacy, size, or regulatory reasons. Documentation of the acquisition protocol and potential confounding factors related to analysis, provenance of the individual annotations and analysis results, and use of unique identifiers to prevent cross-contamination of testing and training data are important for ensuring quality of the analysis. The use of standards is critical to scale production deployment of federated learning systems. They provide improved consistency of data representation and harmonization of imaging with the analysis results.

Overall, we emphasize the importance of a collaborative approach for addressing challenges in imaging informatics at the national and international levels. Topics such as data standardization, interoperability, and sustainable open-source software development cannot be addressed by an individual group. Open coordinated effort involving a broad range of stakeholders, including clinical researchers, engineers, and industry collaborators, is essential. Joint development of the standard, reference implementations and datasets, and reusable components is ultimately beneficial to both academic and commercial efforts.

Most recently, the BWH team led a consortium of collaborators, including many of the QIICR investigators, who were awarded the contract to establish the NCI Imaging Data Commons (IDC),^50^ a new component of the NCI Cancer Research Data Commons (CRDC). Our IDC approach is motivated by our QIN and QIICR experience, and will use DICOM to harmonize analysis results that IDC will share.

Challenges remain in establishing and maintaining the infrastructure of analysis tools and standardized imaging datasets. Sustainability of open-source tools developed in academia is often problematic, because continuous maintenance effort is required for changes in dependencies and operating systems, user support, and implementation of new features. Our use of 3D Slicer as a delivery platform partially mitigates this issue by leveraging infrastructure, community effort, and contributions from numerous other supporting projects. Development and dissemination of most of the QIICR tools would be extremely challenging without leveraging the 3D Slicer infrastructure. Sustained support for extensible open-source platforms that provide generic reusable components and a marketplace-like infrastructure for new contributions is critical to reduce redundancy, build community, and support tool dissemination and outreach.

Despite significant progress in community adoption of DICOM for QI results, there remains significant effort required to integrate it into academic and commercial tools and workflows. Although improving, support in commonly used tools is limited. Adoption of FAIR (Findable, Accessible, Interoperable, Reusable) data stewardship principles^51^ requires time and sustained community effort to improve the standards, tools, documentation, and outreach. We envision the CRDC IDC as a mechanism and laboratory to refine and demonstrate best practices for curating and interoperating with standardized image and image-derived data, and improve access to QI and AI analysis tools.
